# Quantification of Methylated Selenium, Sulfur, and Arsenic in the Environment

**DOI:** 10.1371/journal.pone.0102906

**Published:** 2014-07-21

**Authors:** Bas Vriens, Adrian A. Ammann, Harald Hagendorfer, Markus Lenz, Michael Berg, Lenny H. E. Winkel

**Affiliations:** 1 Department of Water Resources and Drinking Water, Swiss Federal Institute of Aquatic Science and Technology, Dübendorf, Switzerland; 2 Institute of Biogeochemistry and Pollutant Dynamics, Swiss Federal Institute of Technology, Zürich, Switzerland; 3 Department of Environmental Toxicology, Swiss Federal Institute of Aquatic Science and Technology, Dübendorf, Switzerland; 4 Department of Thin Films and Photovoltaics, Swiss Federal Institute for Material Science and Technology, Dübendorf, Switzerland; 5 Institute for Ecopreneurship, School of Life Science, University of Applied Sciences and Arts Northwestern Switzerland, Muttenz, Switzerland; 6 Department of Environmental Technology, Wageningen University, Wageningen, The Netherlands; CINVESTAV-IPN, Mexico

## Abstract

Biomethylation and volatilization of trace elements may contribute to their redistribution in the environment. However, quantification of volatile, methylated species in the environment is complicated by a lack of straightforward and field-deployable air sampling methods that preserve element speciation. This paper presents a robust and versatile gas trapping method for the simultaneous preconcentration of volatile selenium (Se), sulfur (S), and arsenic (As) species. Using HPLC-HR-ICP-MS and ESI-MS/MS analyses, we demonstrate that volatile Se and S species efficiently transform into specific non-volatile compounds during trapping, which enables the deduction of the original gaseous speciation. With minor adaptations, the presented HPLC-HR-ICP-MS method also allows for the quantification of 13 non-volatile methylated species and oxyanions of Se, S, and As in natural waters. Application of these methods in a peatland indicated that, at the selected sites, fluxes varied between 190–210 ng Se·m^−2^·d^−1^, 90–270 ng As·m^−2^·d^−1^, and 4–14 µg S·m^−2^·d^−1^, and contained at least 70% methylated Se and S species. In the surface water, methylated species were particularly abundant for As (>50% of total As). Our results indicate that methylation plays a significant role in the biogeochemical cycles of these elements.

## Introduction

Selenium (Se) is essential for human health, but the range between beneficial quantities and toxic concentrations of Se is narrow [1]. The element is irregularly distributed over the Earth's surface [2], which leads to uneven Se levels in agronomic produce and consequently within the terrestrial food chain throughout different areas in the world. On a global scale, Se deficiency is more prevalent than dietary Se excess and is associated with a reduced health status in livestock and humans [3]. Insight into the mechanisms that determine the distribution and speciation of Se in surface environments, such as in agricultural soils, is therefore indispensable. The chemical properties of Se are similar to those of sulfur (S) [1], and these two elements often show similar biogeochemical behavior in the environment [4], [5]. Emissions of volatile organic S species are so substantial (e.g., the flux of dimethyl sulfide [DMS] from oceans to the atmospheric is 38–40 Tg·yr^−1^) [6] that they play an important role in the global S cycle. Analogously, emissions of volatile Se species (e.g., dimethyl selenide [DMSe], dimethyl diselenide [DMDSe], and dimethyl selenosulfide [DMSeS]) [4] have been identified in various environments, but global atmospheric Se flux estimates still contain large uncertainties [7]. Like Se, the trace element arsenic (As) can have a deleterious impact on human health, but in contrast to Se, it is not considered an essential element [8], [9]. Similar to Se and S, biogenic methylation and volatilization are known to occur for As [10] (e.g., monomethyl arsine [MMA], dimethyl arsine [DMA], and trimethyl arsine [TMA] have been previously measured in emissions from soils) [11]. Because Se and As often not only occur in association with S, but are also linked to S biogeochemistry in many environments [4], [12], [13], it is essential to study the biogeochemical cycling and emissions of the trace elements Se and As in conjunction with S.

Challenges in the quantification of biogenically formed alkylated molecules in the field derive both from the reactivity of these species (i.e., sorption, photoreactions, and [redox-] interconversions) and their typically low environmental concentrations [14]–[17]. Atmospheric concentrations of volatile Se and As species (in the ng·m^−3^ range) [18], [19] and S species (in the µg·m^−3^ range) [20] are generally so low that analyte preconcentration is required. The preconcentration of volatile species with conservation of speciation can be achieved via solid sorptives (e.g., cartridges [charcoal- and Tenax tubes], columns or solid-phase micro-extraction [SPME]) [21]–[23], via gas trapping in mineral acids [24], or using cryotrapping (direct for gaseous samples and coupled with a purge-and-trap system for volatile species dissolved in water) [25], [26]. Following preconcentration, the different species are separated (for instance, via gas- or liquid-chromatography) and detected in the laboratory. Extensive reviews of hyphenated preconcentration- and speciation-methods for the quantification of Se [27]–[30], As [28], [29], [31], [32], and S [15], [16] are available in the literature, and a short overview is given in Table S1 in Supporting Information File S1.

Although available preconcentration techniques for the collection of Se, S, or As species are highly sensitive, they are usually laborious because an additional trap elution step in the laboratory is often necessary before speciation analysis can take place. Furthermore, many available techniques cannot be easily deployed in longer field campaigns in remote locations due to limited sample stability and transportation issues [e.g., involving pressurized (cryo-)gas bottles]. Although multi-element detection [e.g., inductively coupled plasma mass spectrometry (ICP-MS)] is increasingly used, few preconcentration methods have been developed for multi-elemental studies [33] (thus requiring the combination of different single-element techniques), and only a few speciation methods target multiple (trace) elements (e.g. As and Se and S) at the same time [33]–[36]. The majority of preconcentration and speciation methods for Se and As have focused on major oxyanions [17], while less attention has been given to combined S-As (thio-arsenates) [37] and Se-S (seleno-sulfides) species [33] or to naturally occurring (volatile) methylated Se and As species [23], [24], [33].

Here, we present a highly sensitive and field-deployable method for the simultaneous quantification of volatile Se and S species and total volatile As in air. The technique, based on the trapping of volatile species in nitric acid [24], targets multiple elements at the same time, and may be combined with a flow-through box system that can be deployed in various environments. Using HPLC-HR-ICP-MS, we show that information on the original gaseous speciation of Se and S can be deduced from the formation of stable and specific non-volatile oxidation products. In addition, we present a second HPLC-HR-ICP-MS method for the direct speciation analysis of non-volatile methylated species and oxyanions of Se, S, and As in ambient waters. The quantification of volatile organic Se and S species in air overlying a natural wetland, as well as the speciation analysis of non-volatile methylated species of Se, S, and As in wetland surface water, show that the preconcentration and speciation methods are sensitive and robust.

## Materials and Methods

### Reagents and chemicals

Standards of volatile methylated Se compounds [dimethyl selenide (DMSe) and dimethyl diselenide (DMDSe)] and S compounds [dimethyl sulfide (DMS) and dimethyl disulfide (DMDS)] were obtained from Sigma-Aldrich, Buchs, Switzerland. Ultrapure HPLC-grade methanol, sodium borohydride (NaBH_4_) and sodium hydroxide (NaOH) were purchased from Alfa Aesar, Zürich, Switzerland. Ultrapure 70% nitric acid (HNO_3_) was obtained from Carl Roth GmbH, Karlsruhe, Germany. The following solutions of non-volatile standards were used in the speciation methods: ICP-MS standards of selenite (Se[IV]), sulfate (S[VI]), and arsenate (As[V]) (J.T. Baker, Avantor, Griesheim, Germany), dissolved sodium selenate (Se[VI]), methane seleninic acid (MSeA), methane sulfonic acid (MSA), dimethylsulfoxide (DMSO), dimethyl sulfone (MSM), sodium (meta)arsenite (As[III]), dimethyl arsonic acid (DMAA) (Sigma-Aldrich, Buchs, Switzerland) and monomethyl arsenic acid sodium salt (MMAA) and trimethyl arsenoxide (TMAO) (Argus Chemicals, Vernio, Italy). Chromatography eluents were prepared with ultrapure ammonium, disodium carbonate (Na_2_CO_3_), and sodium bicarbonate (NaHCO_3_) (Fluka, Sigma-Aldrich, Buchs, Switzerland), ultrapure HNO_3_, and ultrapure methanol. All glassware, tubing, syringes, and vials used in experiments were acid-washed in 1% ultrapure HNO_3_ and rinsed with ultrapure water (18.2MΩ, Thermo, NANOpure, Reinach, Switzerland) before use. All chemicals were analytical grade or higher.

### Gas trapping experiments

**Figure 1:**
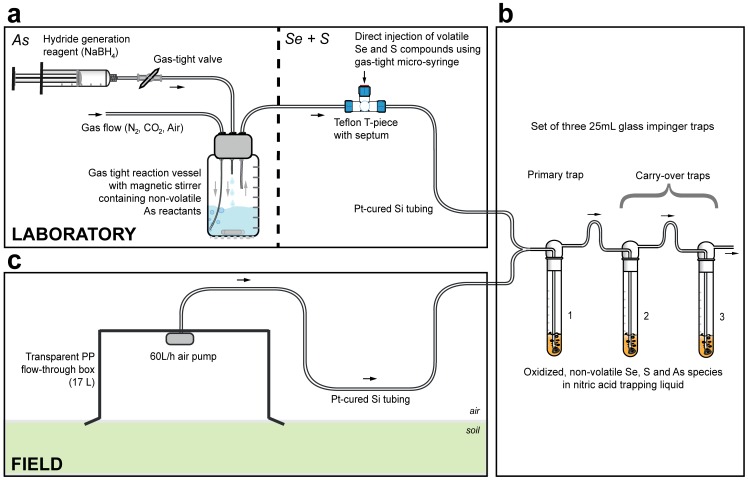
Overview of the experimental set-ups for gas trapping experiments in the laboratory and in the field. (A) Schematic of the experimental set-up for the laboratory gas trapping experiments, with the separate in-situ production of volatile methylated As species in a gas-tight reaction vessel (left) and direct introduction of volatile methylated Se and S species (right), connected to (B), a set of glass impingers filled with concentrated nitric acid and **c**, schematic of the experimental set-up for the field gas trapping experiments, which consists of a flow-through box equipped with an air pump connected to the set of glass impingers (B). During field application, one impinger was connected to one flow-through box and the flow-through boxes were deployed in triplicate.

The trapping efficiency of volatile Se, S, and As compounds (DMSe, DMDSe, DMS, DMDS, MMA, DMA, and TMA) was studied in the laboratory in a gas trapping set-up as shown in Figure 1A,B [24]. Three glass impingers (25 mL, Labo-Tech, Muttenz, Switzerland) were connected in series with Pt-cured Si-tubing (Tygon Masterflex, Thermo Fisher, Reinach, Switzerland). The primary trap and two carry-over traps contained 15 mL ultrapure 70% HNO_3_. A gas stream of N_2_, CO_2_, or air (Air Liquide, Gümlingen, Switzerland) was regulated by a flow-controller (10–100 mL·min^−1^) and guided through the impingers. Between 0.1 and 1 mg of the Se and S target compounds were individually injected directly into the gas stream through a Teflon septum (Swagelok, Arbor AG, Brugg, Switzerland) either undiluted (in case of S) or in dilutions of 1∶100 with ultrapure methanol (in case of Se) using 10 and 100 µL gas-tight micro syringes (Hamilton, Bonaduz, Switzerland).

Volatile As compounds were produced in situ in a 50 mL gas-tight reaction vessel connected in front of the impingers (Figure 1A). The volatile species (MMA, DMA, and TMA) were individually produced by hydride formation from non-volatile reagents (MMAA, DMAA, and TMAO, respectively). For this, between 0.1 and 1 mg of educts were dissolved in 10 mL 1% HNO_3_ in the reaction vessel. After dissolution, the pH was adjusted to 0>pH>3 with dilute NaOH, depending on the targeted volatile As species (the formation of volatile methylated As compounds via hydride generation is greatly pH dependent [38], [39]). The solution was subsequently purged with N_2_ for >20 minutes. The hydride generation process was initiated by the addition of 20 mL 0.5 M borane (NaBH_4_) solution. The strongly acidic conditions and high borane-to-substrate ratios (>1000 times stoichiometric excess of NaBH_4_) result in the fast formation of fully hydrogenated volatile arsines [40], which were transported by the inert gas flow into the gas traps. After each experiment, an aliquot of the reagent mixture was analyzed for remaining As.

Trapping efficiencies were calculated as the ratio between total elemental amounts of analyte in the gas traps and the total elemental amounts of introduced volatile analyte (either via direct injection of Se and S or calculated from the amount of remaining As in the reaction vessel). Before each trapping experiment, a blank from each of the three impingers was analyzed. The weights of the trapping liquids were recorded before and after trapping to account for potential evaporation of the acid. The trapping was continued for 120 minutes, after which the trapping liquids were immediately stored at 4°C in 20 mL acid-washed amber-glass vials with a PTFE cap (BGB Analytics, Boeckten, Switzerland). Subsequent analysis took place within one week after trapping. Potential losses of volatile compounds (e.g., due to diffusion into the tubing) were accounted for by acid-washing (24 h in 50 mL 0.1 M HNO_3_) the tubing and T-piece after the experiments, and analyzing the wash for Se, S, and As.

### Elemental- and speciation analysis of trapped air and surface water

The total elemental concentrations of Se and As in trapping liquids and surface waters were measured using ICP-MS (Agilent 7500cx, Basel, Switzerland) and ICP-OES (Spectro Arcos, Kleve, Germany); total elemental concentrations of S were analyzed by ICP-OES and HR-ICP-MS (Thermo Element 2, Reinach, Switzerland). Details of the total elemental analyses are given in the Supporting Methods and Table S2 in Supporting Information File S1.

Volatile species that were trapped in the nitric acid trapping liquids were analyzed using a HPLC-procedure with gradient elution (henceforth referred to as ‘air-method’). The method was developed specifically for acidic trapping liquid samples, and served to simultaneously separate 11 non-volatile methylated or oxyanionic Se, S, and As species (details can be found in Table S3 in Supporting Information File S1). Chromatographic separation was achieved using a PAX-500 Omnipac mixed-mode column (Dionex, Thermo, Reinach, Switzerland), after which elemental detection took place with HR-ICP-MS. The studied species were identified by retention-time matching as well as by Electrospray-Ionization Tandem Mass Spectrometry (ESI-MS/MS, details in the Supporting Methods in Supporting Information File S1) (Thermo LTQ Orbitrap XL ETD, Reinach, Switzerland) and quantified by peak integration using OriginPro 8 software (OriginLab, Northhampton, MA, USA).

In addition, a second gradient HPLC procedure was developed for circumneutral water samples using the same mixed-mode column (henceforth referred to as ‘water method’). Using this ‘water method’, 13 non-volatile methylated or oxyanionic Se, S, and As species were simultaneously separated. Detection, identification, and quantification were performed as described above (see Table S3 in Supporting Information File S1). Both chromatographic methods used ammonium nitrate (NH_4_NO_3_) as the primary eluent, which has advantages over other eluents [41]. The characteristics of both chromatographic methods are briefly discussed in in Supporting Information File S1.

### Field study

The laboratory-tested chemotrapping method was combined with a flow-through box system and deployed for air collection in *Gola di Lago*, a minerotrophic peatland in southern Switzerland [42] (permission granted by the Department of Environment Ticino, Switzerland). The flow-through boxes (transparent polypropylene boxes, volume 17 L, covered surface area 0.2 m^2^, Iris Ohyama Europe B.V., Tilburg, the Netherlands) were equipped with an air pump (60 L•h^−1^, TetraTec GmbH, Melle, Germany) (see Figure 1C). In a 24 h period of air sampling, three liquid chemotrapping samples were simultaneously collected from different locations (5 meters apart) in the peatland, and three surface water samples were collected from the same locations. The chemotrapping liquid samples and surface water samples were analyzed for their total elemental Se, S, and As concentrations, as well as for the speciation of these elements using the methods described above. One set of the samples was spiked with standards to verify the reproducibility of the chemotrapping method and speciation methods.

## Results and Discussion

### Chemotrapping efficiencies

**Figure 2:**
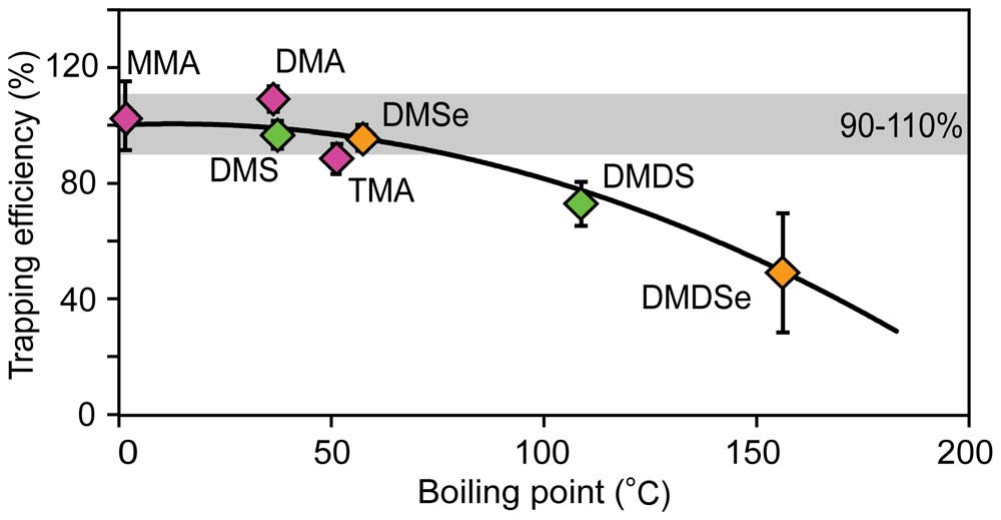
Relationship between the efficiency of chemotrapping in nitric acid and boiling points of the studied volatile compounds. Error bars indicate the standard deviation of the measurements of triplicate samples.

The yield of volatile As species production by hydride generation (calculated from the As concentration remaining in the reaction vessel) was 72±3% for MMA, 91±4% for DMA, and 32±2% for TMA. From these, the total trapping efficiencies were 104% (MMA), 110% (DMA), and 89% (TMA), respectively. An overview of the trapping efficiencies of all the investigated Se, S, and As species is given in Table 1. The standard deviations of the trapping efficiencies were low (<12%), indicating that the total amounts of trapped elements can be reproducibly reconstructed. Carryover into the second and third impingers was minimal, with over 90% of the introduced species trapped in the first impinger (see Table S4 in Supporting Information File S1). Trapping efficiencies were dependent on the degree of volatility of the studied species (Figure 2). For the more volatile compounds (DMS, DMSe, MMA, DMA, and TMA; boiling points ≤57°C), the trapping efficiencies were between 89% and 110%. Less volatile compounds (DMDS and DMDSe; boiling points ≥100°C) showed lower but reproducible trapping efficiencies (between 50% and 74%). Analysis of the acid wash of the T-piece and tubing showed that within a trapping time of 120 min, analytes with a higher boiling point (and lower vapor pressure) were not completely evaporated and transported into the traps, but instead remained partly adsorbed to the tubing. Repeated total elemental and speciation analysis of the undiluted trapping liquids (stored at 4°C for 30 d) yielded similar recoveries (within 95% agreement) and speciation, indicating that the formed species are stable and preserved.

**Table 1:** Studied volatile species, including their structure and boiling points, calculated total trapping efficiencies, and observed reactions products and structures after trapping and transformation in concentrated nitric acid.

Studied species		Boiling point (°C)^a^	Total trapping efficiency (%)^b^	Identified reaction product(s)	
DMSe	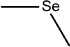	57	96±2	DMSeO	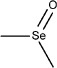
DMDSe	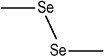	156	50±11	MSeA	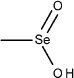
DMS	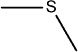	37	101±5	DMSO	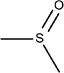
DMDS	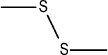	109	74±8	MSA	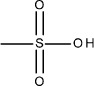
MMA	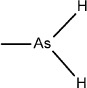	1	104±12	As[V]	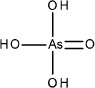
DMA	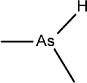	36	110±4	As[V]	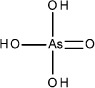
TMA	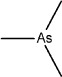	51	89±6	As[V], MMAA	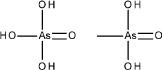

a Boiling point at 1 atm ^b^efficiency using a 30 mL·min^−1^ N_2_ gas flow, summed over three impingers, standard deviation from triplicate experiments.

Abbreviations: dimethyl selenide (DMSe), dimethyl diselenide (DMDSe), dimethyl sulfide (DMS), dimethyl disulfide (DMDS), monomethyl arsine (MMA), dimethyl arsine (DMA), trimethyl arsine (TMA), dimethyl selenoxide (DMSeO), methane seleninic acid (MSeA), dimethyl sulfoxide (DMSO), methane sulfonic acid (MSA), arsenate (As[V]), monomethyl arsonic acid (MMAA).

### Deduction of the gaseous speciation

**Figure 3:**
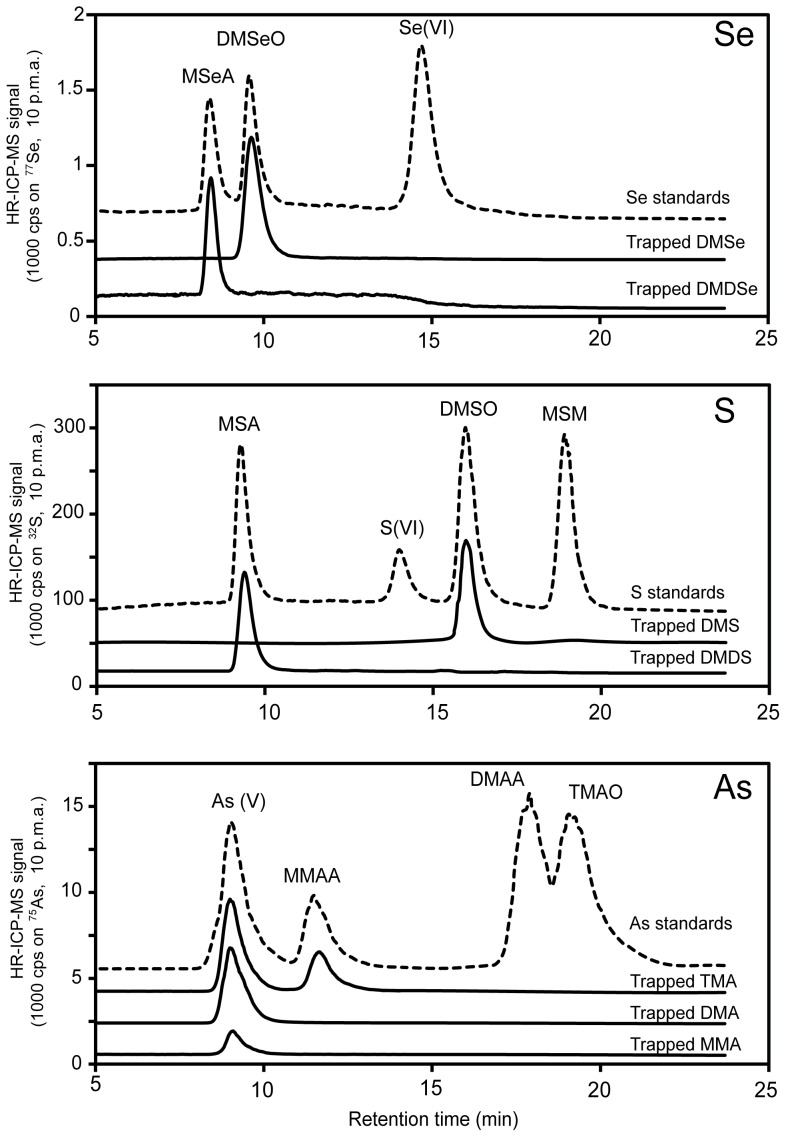
Analysis of trapping liquid samples after gas trapping experiments with volatile, methylated Se, S and As compounds. Stacked chromatograms of solutions with non-volatile Se (top), S (middle), and As (bottom) standards (dashed lines), and chromatograms of diluted nitric acid trapping liquids (solid lines) produced in the gas trapping experiments with volatile organic Se, S and As compounds. The chromatograms are ten-point moving averages.

The trapped volatile Se, S, and As compounds in the nitric acid trapping liquid were investigated with the ‘air method’. In the trapping liquids of the Se and S trapping experiments, single non-volatile transformation products were observed. The peak retention times of the analyzed trapping liquids matched with those of known standards and indicated the formation of DMSO from DMS, MSA from DMDS, DMSeO from DMS, and MSeA from DMDSe upon trapping in nitric acid (Figure 3, Table 1). The identities of these compounds were further confirmed by spiking the trapping liquids with the corresponding known standard, as well as by analyzing the trapping liquids with ESI-MS/MS (see Figure S1 and Table S5 in Supporting Information File S1). The trapping of volatile MMA and DMA both led to the formation of As[V], and TMA trapping resulted in the formation of both As[V] and MMAA (Figure 3), indicating that demethylation occurred during the acid-trapping of these As species. In summary, the trapping method not only enables quantification of the total concentrations of volatile Se, S, and As in air due to the reproducible trapping efficiencies, but also allows for a quantitative reconstruction of the original gaseous speciation of volatile S and Se (but not As) due to the formation of single and exclusive oxidized (non-volatile) trapping products.

The limits of detection (LOD, 3×σ) for the investigated species in the trapping liquids using the HPLC-HR-ICP-MS ‘air method’ were 0.17–0.23 µg·L^−1^ for Se species, 0.27–1.1 µg·L^−1^ for As species, and 2–10 µg·L^−1^ for S species (see Table S3 in Supporting Information File S1). These values translate to lower LODs in the original gas phase due to the preconcentration and accumulation effect [43]. The gas phase LODs of the investigated species are ultimately determined by the instrumental detection limit (<22 pg Se, <65 pg As, and <780 pg S, based on the aqueous concentration and injection volume). Consequently, preconcentration using a 60 L·h^−1^ gas flow for 24 h in 15 mL nitric acid (as applied during the collection of field samples [42]) results in gas-phase LODs of <2.4 pg·L^−1^ for the Se species. Quantification limits may be further improved depending on the applied air flow and the duration of trapping, the purity of the trapping liquid, or instrumentally by using, for example, hydride generation or additional preconcentration techniques prior to ICP-MS [21], [29], [44].

### Emissions from the peatland

**Figure 4:**
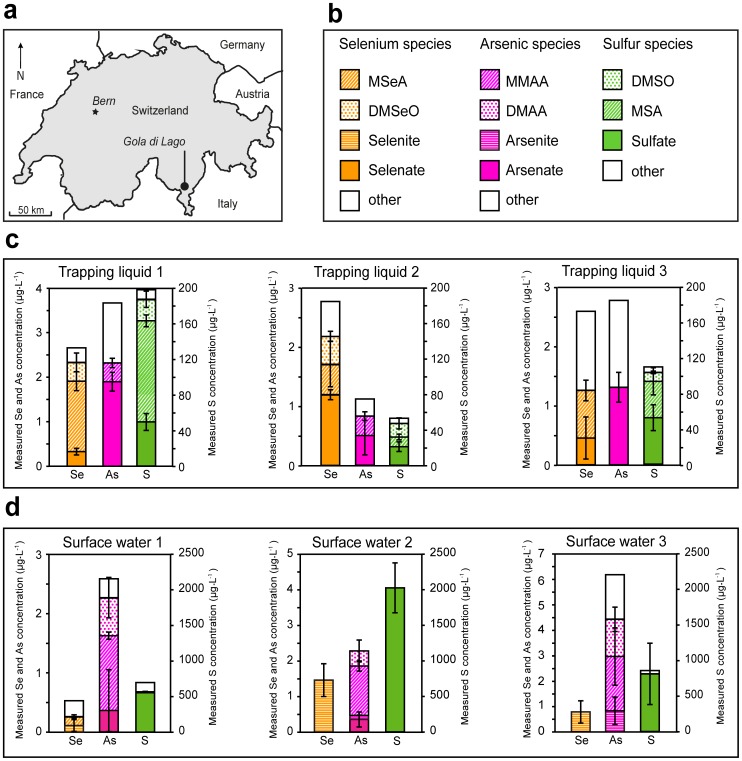
Field study site and speciation analysis of field samples. (A) Location of the studied minerotrophic peatland, Gola di Lago, in southern Switzerland, (B) Legend depicting the investigated species in both the trapping liquids and the surface waters (the fraction of other species was calculated as the total elemental concentration minus the elemental sum of the identified species), (C) Speciation of Se, As and S in the three trapping liquid samples (elemental basis), (D) Speciation of Se, As and S in the three surface water samples (elemental basis). Error bars indicate the standard deviation of triplicate analysis of the samples.

**Figure 5:**
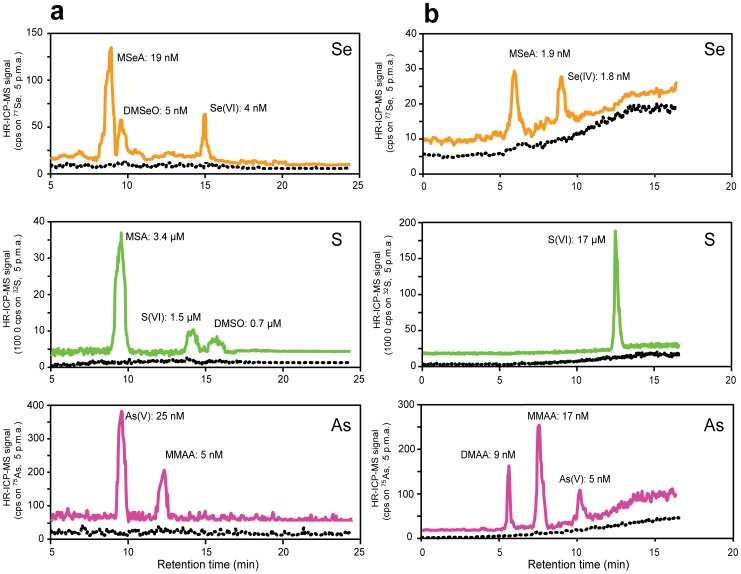
Chromatograms of trapping liquid- and surface water samples collected at Gola di Lago. (A) Stacked chromatogram of the gas trapping liquid sample 1 from Gola di Lago for Se (top), S (middle), and As (bottom) and (B) Stacked chromatogram of the surface water sample 1 from Gola di Lago for Se (top), S (middle), and As (bottom). Chromatograms for blanks are indicated by dashed lines. All chromatograms are five-point moving averages, and the identified compounds and their molar concentrations (on an elemental basis) are indicated at the corresponding peaks. Details of both methods are given in Table S3 in Supporting Information File S1.

Analysis of the trapping liquids that were collected in the field allowed for the quantification of naturally emitted volatile Se and S species, as well as the quantification of total As emissions. In the trapping liquids collected at three different locations, the total concentrations ranged between 2.6–2.8 µg·L^−1^ Se, 1.2–3.7 µg·L^−1^ As, and 53–200 µg·L^−1^ S (Figure 4). Different methylated species of Se (MSeA, DMSeO), S (MSA, DMSO) and As (MMAA) were identified in the trapping liquids, as well as non-methylated anionic Se, As, and S species (an example chromatogram from a field sample is shown in Figure 5A). Spiking of a trapping liquid with corresponding standards yielded >90% recovery at the retention times of the trapped species (see Table S6 in Supporting Information File S1), which indicated that the species were correctly identified and quantified. The high recovery of the spiked standards indicated that matrix effects (e.g., caused by the trapping of other emitted volatiles) were insignificant.

Using the air collection rate and flow-through box surface [42], the total elemental emissions at the three locations in the peatland were calculated to vary between 190–210 ng Se·m^−2^·d^−1^, 90–270 ng As·m^−2^·d^−1^, and 4–14 µg S·m^−2^·d^−1^. According to the conversion of trapped species as validated in the laboratory experiments (Table 1), the observed methylated species in the trapping liquids corresponded with the emission of 19–56 ng DMDSe·m^−2^·d^−1^, 0–34 ng DMSe·m^−2^·d^−1^, 0.5–5.7 µg DMDS·m^−2^·d^−1^, and 0.7–1.6 µg DMS·m^−2^·d^−1^ at the three locations in the peatland, which is comparable to fluxes observed in a larger field campaign in the same peatland [42]. The presence of a methylated As species in the trapping liquids (0–0.4 µg·L^−1^ MMAA) indicated the volatilization of methyl-As compounds, even though the exact original gaseous speciation of As cannot be reconstructed. Comparing the sum of all identified species (elemental basis) with measured total elemental quantities in the trapping liquids, up to 85% of total Se, up to 76% of total As, and up to 94% of total S was identified in the three natural air samples (Figure 4, see also Table S6 in Supporting Information File S1). Unidentified species were present in all trapping liquids, which may have been caused by the emission of other volatile species that were not specifically investigated in this study (e.g., hydrogen sulphide [H_2_S], carbonyl sulphide [COS], methane thiol [CH_3_-S-H] carbon disulphide [CS_2_] [14], and Se analogues [7]). The incorporation of these additional species in the presented trapping technique could be a next step in the further development of this method.

### Aqueous speciation of the peatland surface water

The ‘water method’ allowed for the simultaneous separation of 13 non-volatile, methylated and oxyanionic Se, S and As compounds in natural waters (see Figure S2 in Supporting Information File S1). The ‘water method’ also included the species Se[IV] and As[III] because in ambient, slightly reducing waters (but not in the oxidative nitric acid trapping liquid) Se[IV] and Se[VI] or As[III] and As[V] may coexist [1], [45]. The limits of detection for the species investigated in the ‘water method’ are 0.10–0.17 µg·L^−1^ for Se species, 0.16–0.31 µg·L^−1^ for As species, and 16–22 µg·L^−1^ for S species (see Table S3 in Supporting Information File S1), which is comparable to the ‘air method’ and in the same order of magnitude as previously reported LODs of ICP-MS-based speciation methods (see Table S1 in Supporting Information File S1).

The peatland surface water samples showed considerable variation in total elemental concentrations on a small spatial scale (Figure 4). Compared to the speciation of Se and S in the trapping liquids, the aqueous speciation of Se, S, and As at the three investigated locations was relatively uniform. All analyzed surface water samples contained only sulfate (between 546–2002 µg·L^−1^) as an identified S species, while both anionic and methylated species of Se (0.1–1.5 µg·L^−1^ Se[IV] and 0–0.2 µg·L^−1^ MSeA) and As (0–0.4 µg·L^−1^ As[V], 0–0.8 µg·L^−1^ As[III], 1.3–2.1 µg·L^−1^ MMAA, and 0.5–1.5 µg·L^−1^ DMAA) were identified at the three locations (Figure 4 and Figure 5B). Aqueous methylated species were thus not so relevant for Se and S, but appeared to be major species for As. Even though methylated As species are typically not the most abundant species in natural surface waters [46], their presence has been previously reported [47].

Spiking of a surface water sample with known standard solutions yielded >90% recoveries for all compounds (see Table S6 in Supporting Information File S1), confirming the species identities and illustrating that matrix effects were insignificant. In surface water sample 2, the sum of the identified species equaled the total elemental concentrations, (Figure 4) while in the other two surface water samples, small amounts of other species could not be identified (up to 0.3 µg·L^−1^ of Se, 1.7 µg·L^−1^ of As, and 140 µg·L^−1^ of S). Polysulfides and combined S-Se and S-As species have been previously identified as potentially relevant species in the environment (e.g., thio-arsenates [37]). However, no simultaneous As-S, Se-S, and As-Se peaks were observed, suggesting that combined species have already transformed during sampling, or were not major constituents of the collected surface water samples. Dissolved volatile species are known to yield higher responses in ICP-MS due to their more efficient vaporization and transport into the plasma compared to non-volatile calibration standards [48]. Subsequent overestimation of the total elemental concentration may be another explanation for its poor agreement with the elemental sum of the identified species. Indeed, dissolved volatile species of Se and S were identified in the surface water of Gola di Lago [SPME-GC-MS analysis of the surface water indicated around 10 and 100 ng·L^−1^ DMSe and DMS, respectively (data not shown)].

## Conclusions

Laboratory validation experiments and field-testing demonstrate that the presented air trapping method for volatile species of Se, S, and As in nitric acid is a reliable preconcentration method for the determination of their total emissions in different contexts. The deduction of the original gaseous speciation for Se and S compounds is possible via the formation of specific transformation products and the gaseous As speciation may be qualified using existing techniques [23], [39]. To circumvent potential hazards during handling (nitric acid), the used quantities of acid can be kept small (<15 mL). In addition, acid traps may be prepared in the laboratory and subsequently transported to the field for installation. Important advantages of gas trapping in nitric acid over other preconcentration methods [15]–[17] include the ease and cost of operation (e.g., no pressurized gas bottles are required and the direct, on-site preconcentration eliminates the need for additional sample preparation such as trap elution) and the storability of the samples (i.e., sample concentration and speciation are stable for at least one month).

A considerable variation in the concentration and speciation of Se, S, and As was observed in the studied peatland, indicating that methylation and volatilization were highly variable on small spatial scales (within meters). Quantification of species in the aqueous and gaseous phase indicated that emissions of methylated species differ significantly per element and that the underlying chemical pathways may be more complex than is often assumed. For instance, DMSe is usually referred to as the main volatilized Se species [7], but up to 40% of all trapped Se species in this study did not appear to originate from the latter.

Our speciation measurements were conducted using HPLC coupled to HR-ICP-MS, but the two presented speciation methods may also be coupled to other (or multiple parallel) detection techniques (e.g., atomic emission spectrometry for S or atomic fluorescence/adsorption spectroscopy for As and Se). Complemented by the presented chemotrapping method, these techniques can help to better understand the mechanisms of methylation and volatilization of Se, S, and As in various natural environments. The combination of the gas trapping method with species separation and with sensitive multi-element detection opens up possibilities for studying the emissions of other trace elements that undergo alkylation and volatilization in natural systems (e.g., the halogens Cl, Br, and I, and the trace elements Sb, Te, and Bi) [49]. Additional potential applications include research on natural emissions from the marine environment (e.g., methyl-halogen emissions), as well as monitoring of industrial emissions (e.g., emissions (of alkylated species) from industrial sites and from wastewater treatment plants).

## Supporting Information

File S1 File containing Figures S1–S2, Tables S1–S6, Supporting Discussion, Supporting Methods, and Supporting References.
(DOCX)
