# Supplementary material for: Quantification of Methylated Selenium, Sulfur, and Arsenic in the Environment
Source: PLoS One. 2014 Jul 21;9(7):e102906. doi: 10.1371/journal.pone.0102906 (PMC4105483; doi:10.1371/journal.pone.0102906)
Supplement: File S1 — File containing Figures S1–S2, Tables S1–S6, Supporting Discussion, Supporting Methods, and Supporting References. (DOCX) [file pone.0102906.s001.docx]

Quantification of methylated selenium, sulfur, and arsenic in the environment

Bas Vriens, Adrian A. Ammann, Harald Hagendorfer, Markus Lenz, Michael Berg, and Lenny H.E. Winkel

Supporting Information File S1

Figures S1–S2

Tables S1–S6

Supporting Discussion

Supporting Methods

Supporting References

Edited for publication in *PLOS ONE*, 29.06.2014

**
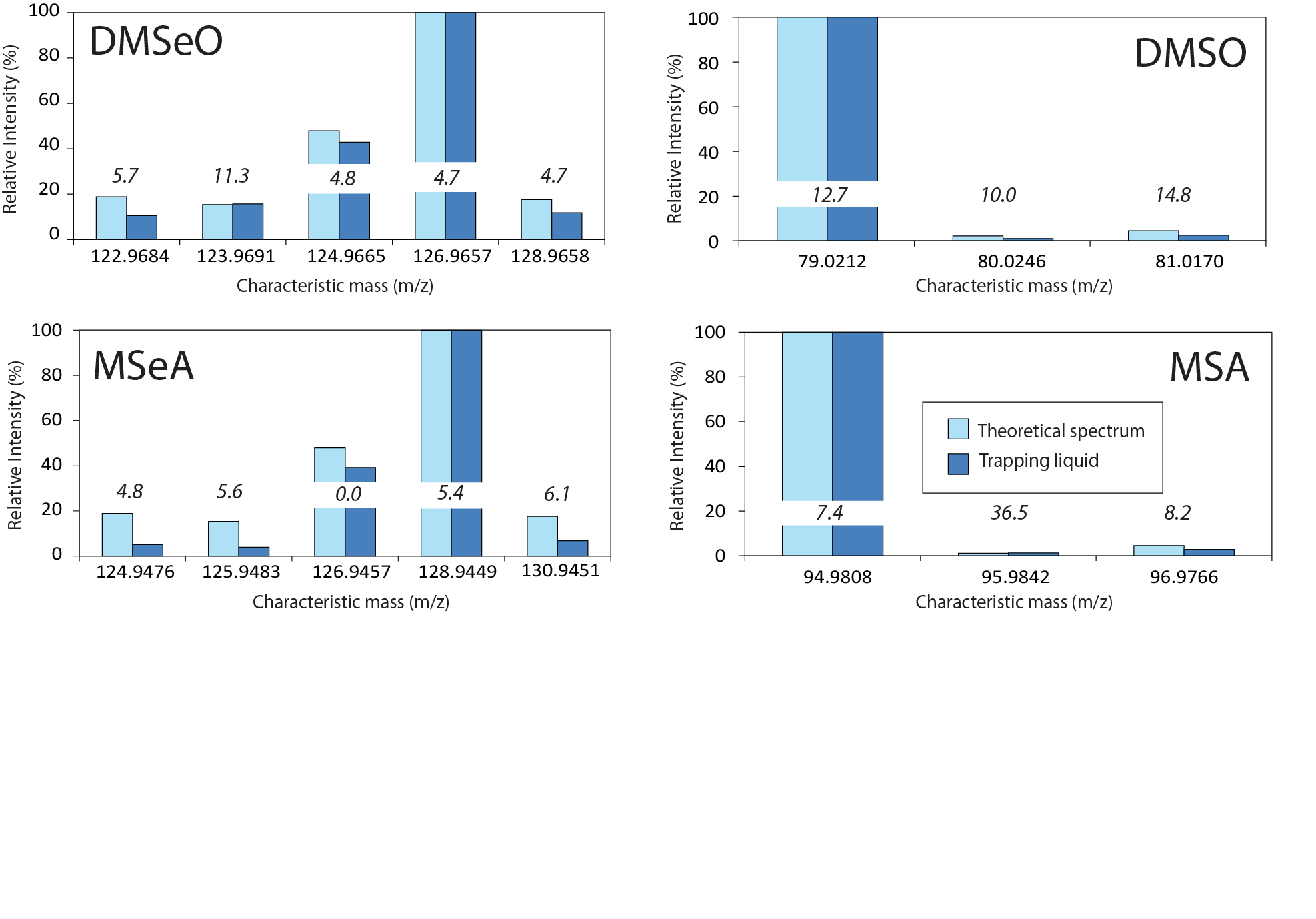
**

**Figure S1.** **Comparison of theoretical mass spectra with measured mass spectra of DMSeO, DMSO, MSeA, and MSA in trapping liquid samples using ESI-MS/MS.**

Relative MS intensities (y-axis) are shown for the characteristic masses (x-axis) of each of the investigated species (legend in MSA-frame). Numbers in italics indicate the absolute deviation from the characteristic mass Δ m/z (ppm). Experimental details are provided in the Supporting Methods in Supporting Information File S1.

*
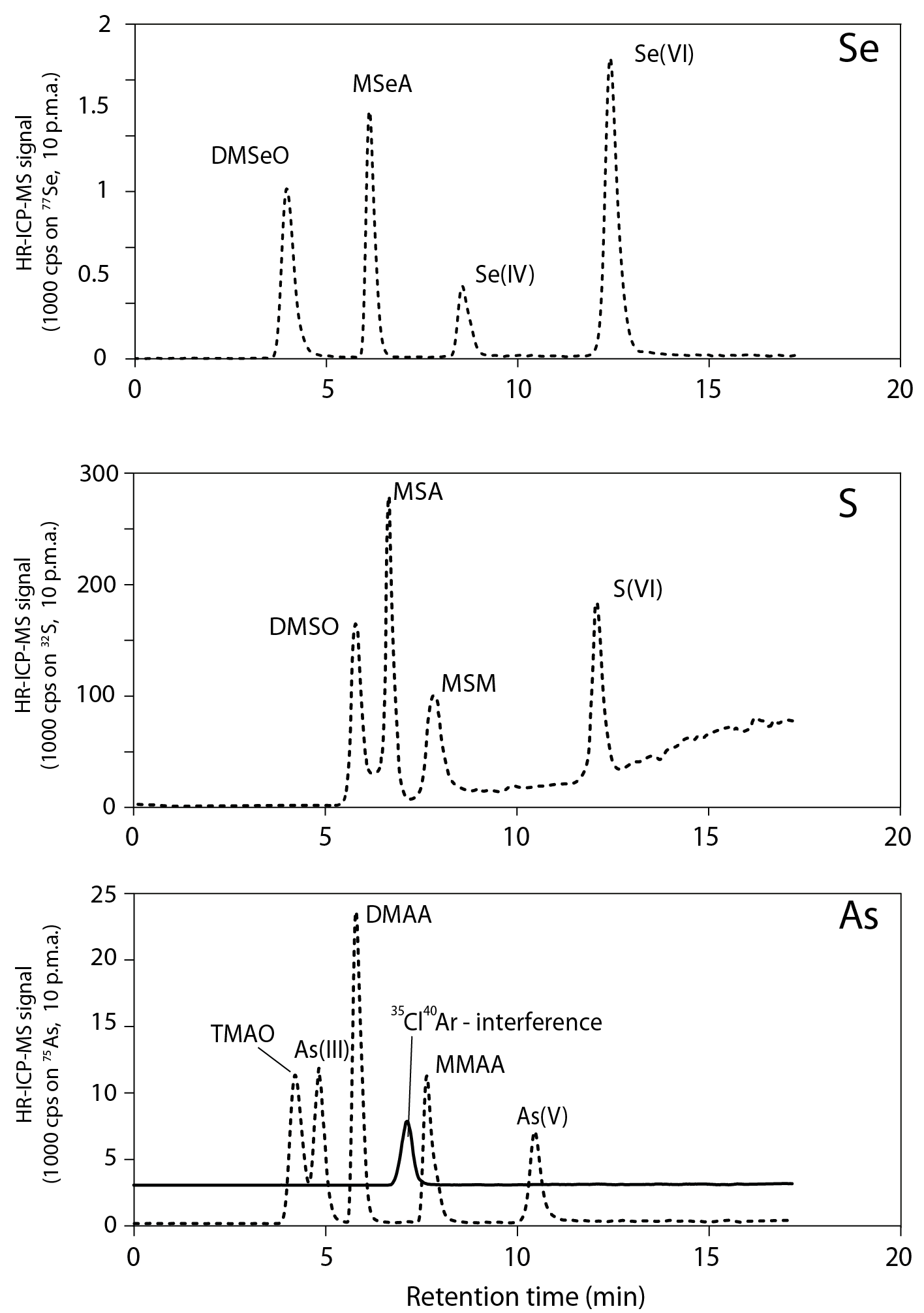
*

**Figure S2. Chromatograms of non-volatile, aqueous Se, S and As species using the ‘water method’.**

The stacked chromatograms illustrate the simultaneous elution of non-volatile Se (top), S (middle), and As (bottom) species (dashed lines) and the potential ^35^Cl^40^Ar interference (solid line) using the ‘water method’. The chromatograms are ten-point moving averages. Experimental details are provided in Table S3 in Supporting Information File S1.

**Table S1.** Selected analytical techniques for the (preconcentration and) quantification of various (non-)volatile Se, S, As species (or combinations thereof) in the gaseous or aqueous phase and their corresponding detection limits (deviating units are indicated if applicable).

| Reference | Method | | Target species | | Phase | | LOD (μg·L^–1^) |  |
| --- | --- | --- | --- | --- | --- | --- | --- | --- |
| Selenium | | | | | | | |  |
| [1] | PT^a^-cryo-AFS | | Volatile Se | | Gaseous | | 4.4 pg·L^–1^ |  |
| [2] | MSPE^b^ -HPLC-ICP-MS | | Inorganic Se & Se-aminoacids | | Aqueous | | 0.025 – 0.149 |  |
| [3] | GC-MIP^c^-AES | | Volatile Se | | Gaseous | | 0.003 – 0. 4 |  |
| [4] | SPME-GC-AES | | (In)organic Se | | Gaseous | | 0.005 – 0.01 |  |
| [5] | FISM^d^-ETAAS | | Inorganic Se | | Aqueous | | 0.005 |  |
| [6] | GC-MIP-AES | | Inorganic Se | | Aqueous | | 0.008 |  |
| [7] | SPE-ICP-MS | | Se-amino acids | | Aqueous | | 0.021 – 0.024 |  |
| [8] | HPLC-UV-HG-AFS | | Inorganic Se & Se-aminoacids | | Aqueous | | 0.02 – 0.05 |  |
| [9] | SPE-ICP-MS | | Inorganic Se & Se-aminoacids | | Aqueous | | 0.045 – 0.21 |  |
| [10] | HPLC-ICP-MS | | Inorganic Se & Se-aminoacids | | Aqueous | | 0.1 – 1.5 |  |
| [11] | HLPC-ICP-MS | | Volatile Se | | Aqueous | | 0.6 – 1.3 |  |
| [12] | HPLC-ICP-MS | | Inorganic Se & Se-aminoacids | | Aqueous | | 0.6 –1.5 |  |
| [13] | HPLC-UV-HG-AFS | | Inorganic Se & Se-aminoacids | | Aqueous | | 1 – 3 |  |
| [14] | HPLC-MW^e^-HG-ICP-MS | | Inorganic Se & Se-aminoacids | | Aqueous | | 1.0 – 5.3 |  |
| [15] | HPLC-ICP-AES | | Se-amino acids | | Aqueous | | 2 – 10 |  |
| [16] | HG-AFS | | Total Se | | Aqueous | | 1 – 5 |  |
| Sulfur | | | | | | | |  |
| [17] | SPME-GC-PFD^f^ | | Volatile organic S | | Gaseous | | 0.01 – 0.36 |  |
| [18] | PT-cryo-GC-FPD^g^ | | Volatile organic S | | Gaseous | | 0.2 – 1 ng·L^–1^ |  |
| [19] | PT-GC-MW-AES | | Volatile organic S | | Gaseous | | 0.4 – 0.9 ng·L^–1^ |  |
| [20] | IC-UV-VIS | | Inorganic S | | Aqueous | | 0.34 |  |
| [21] | CSSWV^h^-HPLC-ICP-MS | | Inorganic S, Organic S | | Aqueous | | 1.7 – 0.17 |  |
| [22] | GC-FID^i^, MIMS^j^ | | Volatile organic S | | Aqueous | | 0.1 – 20 |  |
| [23] | IC-DRC^k^-ICP-MS | | Inorganic S | | Aqueous | | 3.6 – 4.6 |  |
| [24] | IC-ICP-MS | | Inorganic S | | Aqueous | | 35 – 270 |  |
| [25] | SPME-GC-PFD | | Volatile organic S | | Gaseous | | 0.002 – 0.03 |  |
| **Table S1, continued** | | | | | | | |  |
| Reference | Method | Target species | | Phase | | LOD (μg·L^–1^) | | |
| Arsenic | | | | | | | | |
| [26] | HPLC-ICP-MS | Inorganic As, Methylated As | | Aqueous | | 0.005 – 0.01 | | |
| [27] | HPLC-ICP-MS | Inorganic As, Methylated As | | Aqueous | | 0.1 – 0.3 | | |
| [28] | HPLC-UV-VIS | Inorganic As | | Aqueous | | 400 – 1000 | | |
| [29] | IC-ICP-MS | Inorganic As, Organic As | | Aqueous | | 0.008 – 0.024 | | |
| [30] | IC-ICP-MS | Inorganic As, Methylated As | | Aqueous | | 0.1 – 0.75 | | |
| [31] | IC-ICP-MS | Inorganic As, Methylated As | | Aqueous | | 0.1 – 0.3 | | |
| [32] | HPLC-ICP-MS | Inorganic As, Methylated As | | Aqueous | | 0.044 | | |
| [33] | ICP-MS | Inorganic As | | Aqueous | | 0.021 | | |
| [34] | SPME-GC-MS | Methylated As | | Gaseous | | 0.1 μg/m^3^ | | |
| [35] | GC-ICP-MS | Methylated As | | Gaseous | | 20-100 pg | | |
| Combined Se-S-As | | | | | | | | |
| This study | HPLC-HR-ICP-MS | Inorganic and organic Se, S and As | | Aqueous | | 0.13 – 0.23 (Se)  0.16 – 1.1 (As)  2 – 32 (S) | | |
| Combined Se-As | | | | | | | | |
| [36] | HPLC-ICP-MS | Inorganic Se and As, Methylated As | | Aqueous | | 0.006 – 0.4 (As) and 1 (Se) | | |
| [37] | HPLC-HG-AAS | Inorganic Se and As | | Aqueous | | 2 – 20 | | |
| [38] | HPLC-ICP-MS | Inorganic Se and As, Se-aminoacids and methylated As | | Aqueous | | 0.080 – 0.180 | | |
| [39] | IPRP^l^-ICP-MS | Inorganic Se and As, Se-aminoacids and methylated As | | Aqueous | | 20 – 30 (As)  300 – 400 (Se) | | |
| [40] | GC-ICP-MS | Methylated As and Se | | Gaseous | | 21-26 pg/m^3^ | | |
| [41] | GC-ICP/EII^m^-MS | Methylated As and Se | | Gaseous | | - | | |
| Combined Se-S | | | | | | | | |
| [42] | SPME-GC-AES | Volatile S and Se | | Aqueous | | 0.008 | | |
| [43] | SPME-GC-(ICP)-MS | Volatile S and Se | | Gaseous | | 1-10 ppt (Se)  30- 300 ppt (S) | | |

^a^ Purge and Trap, ^b^ Magnetic Solid Phase Extraction, ^c^ Microwave Induced Plasma, ^d^ Flow Injection Microcolumn Separation, ^e^ Microwave, ^f^ Pulsed Flame Photometric Detection, ^g^ Flame Photometric Detection, ^h^ Cathodic Stripping Square Wave Voltammetry, ^i^ Flame Ionization Detector, ^j^ Membrane Introduction Mass Spectrometry, ^k^ Dynamic Reaction Cell, ^l^ Ion Pairing Reversed Phase, ^m^ Electron Impact Ionization

**Table S2.** Settings and analytical characteristics for the total Se and As measurements with ICP-MS, ICP-OES, and HR-ICP-MS.

| ICP-MS (Agilent 7500cx) | |
| --- | --- |
| Collision-reaction cell | Quadrupole, with He or H_2_ |
| Tubing | PEEK and Tygon |
| Tuning | Daily. 10 ppb Li, Co, Y, Ce, and Te in HCl-HNO_3_ |
| Nebulizer | AR 35-1 MicroMist |
| Spray chamber & torch | Scott type, quartz |
| Cones | Ni/Cu |
| Plasma power | 1500 W |
| Internal standard | 1 ppm Sc and 0.1 ppm In and Lu in 1% HNO_3_ |
| Wash solution | 2% HNO_3_ |
| Target masses | ^76^Se, ^77^Se, ^78^Se, ^80^Se, ^75^As |
| Limits of detection^a^ | 2.1 ng Se·L^–1^, 3.6 ng As·L^–1^ |
| ICP-OES (Spectro Arcos) | |
| Tubing | Tygon |
| Tuning | Daily. Spectro I-CAL solution and 2ppm As, Mn, and Pb in 1% HNO_3_ |
| Plasma power | 1300 W |
| Nebulizer | 510-20-Modified Lichte MSDN |
| Spray chamber & torch | Cyclonic, Scott type, quartz |
| Cones | Ni/Cu |
| Internal standard | Rh 343.489 nm |
| Wash solution | 1% HNO_3_ |
| Analytical lines | Se: 196.090 and 204.050 nm |
|  | S: 166.668, 180.731 and 182.034 nm |
|  | As: 189.042 and 193.758 nm |
| Limits of detection^a^ | 5.4 μg Se·L^–1^, 10 μg S·L^–1^, 7.1 μg As·L^–1^ |
| HR-ICP-MS (Thermo Element 2) | |
| Resolution | Medium |
| Tubing | PEEK |
| Nebulizer | PFA MicroFlow, (Elemental Scientific Instrumentation, Omaha, US) |
| Spray chamber | Scott type, quartz, and Peltier-cooled (4°C) |
| Cones | Ni/Cu |
| Tuning | Daily. 1 ppb Sc, Rh, In, U, Y and Lu, and 5 ppb Li and Ba in 1% HNO_3_ |
| Mass calibration | Daily. ^103^Rh |
| Plasma power | 1250 W |
| Wash solution | 1% HNO_3_ |
| Target masses | ^32^S (31.9715 amu), ^34^S (33.9673 amu),  ^77^Se (76.9194 amu), ^78^Se (77.9168 amu),  ^75^As (74.9211 amu) |
| Mass window | 100%, >10 scans per peak |
| Limits of detection^a^ | 0.1 μg Se·L^–1^, 1 μg S·L^–1^, 70 ng As·L^–1^ |

^a^ Three times standard deviation σ

**Table S3.** Settings and characteristics of the ‘air method’ (left) and the ‘water method’ (right) with corresponding analyte retention factors and figures of merit for the investigated analytes.

|  | ‘Air method’^a^ | | | | |  | ‘Water method’^b^ | | | | | |
| --- | --- | --- | --- | --- | --- | --- | --- | --- | --- | --- | --- | --- |
| Flow rate | 500 µL·min^–1^ | | | | |  | 1000 µL·min^–1^ | | | | | |
| Columns | Pax-500 OmniPac Guard (50 × 4mm)  Pax-500 OmniPac mixed-mode (250 × 4mm) | | | | |  | Pax-500 OmniPac Guard (50 × 4mm)  Dionex Gradient-Mixer 4 (50 × 2mm)  Pax-500 OmniPac mixed-mode (250 × 4mm) | | | | | |
|  | | | | | | | | | | | | |
| Gradients | Time  (min) | Eluent A  30mM NO_3_NH_4_,  1% methanol,  pH 7.5 | | Eluent B  50mM Na_2_CO_3_/NaHCO_3_  25% methanol,  pH 8.5 | |  | Time (min) | Eluent A  30mM NO_3_NH_4_,  1% methanol,  pH 7.5 | | | Eluent C  water,  pH 8.4 | |
|  | 0 | 100% | | 0% | |  | 0 | 0% | | | 100% | |
|  | 1 | 100% | | 0% | |  | 1.5 | 0% | | | 100% | |
|  | 10 | 0% | | 100% | |  | 10 | 80% | | | 20% | |
|  | 15 | 0% | | 100% | |  | 11 | 100% | | | 0% | |
|  | 16 | 100% | | 0% | |  | 12 | 0% | | | 100% | |
|  | 25 | 100% | | 0% | |  | 25 | 0% | | | 100% | |
|  | | | | | | | | | | | | |
| Species | Retention factor (k)^c^ (%RSD) | | Linear range (µg·L^–1^) | R^2^ of linear fit | LOD ^d^  (µg·L^–1^) |  | Retention factor (k)^c^ (%RSD) | | Linear range (µg·L^–1^) | R^2^ of linear fit | | LOD ^d^  (µg·L^–1^) |
| Se[VI] | 1.00 (±3.0%) | | 1-100 | 0.976 | 0.18 |  | 2.16 (±3.6%) | | 5-650 | 0.997 | | 0.17 |
| Se[IV] | N.D. | |  |  |  |  | 1.19 (±3.6%) | | 1-250 | 0.978 | | 0.10 |
| MSeA | 0.13 (±3.3%) | | 1-500 | 0.999 | 0.17 |  | 0.55 (±2.6%) | | 1-150 | 0.948 | | 0.15 |
| DMSeO | 0.39 (±3.0%) | | 1-150 | 0.995 | 0.23 |  | 0.02 (±3.7%) | | 5-300 | 0.996 | | 0.13 |
| As[V] | 0.2 (±5.2%) | | 2-200 | 0.965 | 0.36 |  | 1.72 (±3.8%) | | 1-300 | 0.999 | | 0.31 |
| As[III] | N.D. | |  |  |  |  | 0.27 (±3.8%) | | 5-1200 | 0.999 | | 0.20 |
| MMAA | 0.54 (±5.1%) | | 1-100 | 0.983 | 0.27 |  | 0.99 (±3.6%) | | 1-350 | 0.999 | | 0.22 |
| DMAA | 1.37 (±2.7%) | | 2-200 | 0.926 | 1.1 |  | 0.50 (±2.7%) | | 5-450 | 0.999 | | 0.17 |
| TMAO | 1.55 (±3.7%) | | 2-200 | 0.921 | 1.1 |  | 0.12 (±4.5%) | | 2-250 | 0.995 | | 0.16 |
| S[VI] | 0.86 (±2.0%) | | 20-2000 | 0.980 | 10 |  | 2.08 (±3.0%) | | 100-10000 | 0.971 | | 22 |
| MSA | 0.26 (±2.7%) | | 50-1000 | 0.977 | 2 |  | 0.66 (±2.5%) | | 100-10000 | 0.998 | | 17 |
| DMSO | 1.12 (±2.5%) | | 50-7500 | 0.998 | 8 |  | 0.38 (±4.5%) | | 30-3000 | 0.996 | | 16 |
| MSM | 1.55 (±3.3%) | | 50-500 | 0.956 | 13 |  | 0.99 (±4.6%) | | 50-500 | 0.995 | | 32 |

^a^ 2% HNO_3_ sample matrix of the trapping liquids, ^b^ circumneutral sample matrix of natural waters, ^c^ k = (t–t_0_)/t_0,_ with corresponding relative standard deviation σ, ^d^ Limit of detection (LOD, 3 × standard deviation σ).

Abbreviations: not determined (N.D.), selenate (Se[VI]), selenite (Se[IV]), methane seleninic acid (MSeA), dimethyl selenoxide (DMSeO), arsenate (As[V]), arsenite (As[III]), monomethyl arsonic acid (MMAA), dimethyl arsonic acid (DMAA), trimethyl arsine oxide (TMAO), sulfate (S[VI]), methane sulfonic acid (MSA), dimethyl sulfoxide (DMSO), dimethylsulfone (MSM).

**Table S4.** Trapping efficiencies of volatile, methylated Se, S, and As compounds in concentrated nitric acid.

|  | Trapping efficiency (%)^a^ | | | |
| --- | --- | --- | --- | --- |
| Species | First impinger | Second impinger | Third impinger | Sum |
| DMSe | 95.5 ± 1.4 | 0.2 ± 0.2 | 0.0 ± 0.0 | 95.7 ± 1.6 |
| DMDSe | 48.9 ± 10.6 | 0.8 ± 0.1 | 0.7 ± 0.0 | 50.4 ± 10.7 |
| DMS | 96.7 ± 4.7 | 3.4 ± 0.3 | 0.6 ± 0.1 | 100.7 ± 5.1 |
| DMDS | 72.8 ± 7.6 | 0.6 ± 0.5 | 0.6 ± 0.1 | 74.0 ± 8.2 |
| MMA | 103.3 ± 11.9 | 0.1 ± 0.0 | 0.3 ± 0.2 | 103.7 ± 12.1 |
| DMA | 109.0 ± 3.6 | 0.7 ± 0.2 | 0.1 ±0.6 | 109.8 ± 4.4 |
| TMA | 88.4 ± 5.2 | 0.9 ±0.4 | 0.0 ±0.1 | 89.3 ± 5.7 |

^a^ Standard deviations from triplicate experiments. The trapping experiments were conducted using a 30 mL·min^–1^ N_2_ gas flow and 15 mL concentrated nitric acid as the trapping liquid.

Abbreviations: dimethyl selenide (DMSe), dimethyl diselenide (DMDSe), dimethyl sulfide (DMS), dimethyl disulfide (DMDS), monomethyl arsine (MMA), dimethyl arsine (DMA), trimethyl arsine (TMA).

**Table S5.** Identification of trapping products using tandem mass spectrometry. Listed are the observed mass fragments, their relative intensities and the composition of the fragments. Mass fragments of DMSO were not determined (N.D.).

|  | **DMSeO** |  |  | **DMSO** | |  |
| --- | --- | --- | --- | --- | --- | --- |
| Mass fraction (amu) | Relative Intensity (%) | Composition | Mass fraction (amu) | Relative Intensity (%) | Composition | |
| 96.9179 | 100 | HOSe |  |  |  | |
| 94.9386 | 69.7 | CH_3_Se |  | N.D. |  | |
| 111.9414 | 61.43 | CH_4_OSe |  |  |  | |
| 93.9308 | 51.73 | CH_2_Se |  |  |  | |
|  |  |  |  |  |  | |
|  | **MSeA** |  |  | **MSA** |  | |
| Mass fraction (amu) | Relative Intensity (%) | Composition | Mass fraction (amu) | Relative Intensity (%) | Composition | |
| 110.9336 | 100 | CH_3_OSe | 94.9811 | 100 | CH_3_O_3_S | |
| 95.91 | 24.53 | OSe | 79.9579 | 56.27 | O_3_S | |
| 113.9206 | 13.94 | H_2_O_2_Se | 94.9814 | 37.15 | CH_3_O_3_S | |
| 128.9443 | 3.36 | CH_5_O_2_Se | 73.3652 | 12.88 | ? | |

**Table S6.** Measured concentrations, added spikes, and the spike recoveries of the investigated non-volatile Se, S, and As species in trapping liquid sample 1 (left) and in natural water sample 1 (right).

|  | Trapping liquid 1 | | | | Surface water 1 | | | |
| --- | --- | --- | --- | --- | --- | --- | --- | --- |
| Species | Measured concentration (μg·L^–1^) | | Added spike ^a^ (μg·L^–1^) | Spike recovery ^b^ (%) | Measured concentration (μg·L^–1^) | | Added spike ^a^ (μg·L^–1^) | Spike recovery ^b^ (%) |
| Selenate | 0.31 ± 0.07 | | 3.75 | 100 | <LOD | | 100 | 106 |
| Selenite | N.D. | |  |  | 0.14 ± 0.10 | | 2.75 | 102 |
| Sulfate | 47 ± 9 | | 75 | 93 | 546 ± 7 | | 100 | 93 |
| Arsenate | 1.9 ± 0.2 | | 10 | 101 | 0.35 ± 0.69 | | 5 | 103 |
| Arsenite | N.D. | |  |  | <LOD | | 20 | 92 |
| MSeA | 1.5 ± 0.2 | | 22.5 | 101 | 0.15 ± 0.03 | | 5 | 107 |
| DMSeO | 0.4 ± 0.2 | | 3.75 | 99 | <LOD | | 15 | 104 |
| MSA | 109.0 ± 6.3 | | 50 | 102 | <LOD | | 27.5 | 104 |
| DMSO | 23.6 ± 8.7 | | 100 | 93 | <LOD | | 50 | 95 |
| MMAA | 0.4 ± 0.1 | | 10 | 90 | 1.26 ± 0.06 | | 5 | 108 |
| DMAA | <LOD | | 5 | 103 | 0.64 ± 0.34 | | 5 | 101 |
| TMAO | <LOD | | 5 | 101 | <LOD | | 50 | 96 |
|  | | | | |  | | | |
| Total Se ^*^ | | 2.6 ± 0.1 | | |  | 0.60 ± 0.05 | | |
| Total S ^*^ | | 197 ± 3 | | |  | 702 ± 49 | | |
| Total As ^*^ | | 3.7 ± 0.1 | | |  | 2.62 ± 0.11 | | |
|  | |  | | |  |  | | |
| Identified Se ^**^ | | 85% ± 26% | | |  | 48% ± 23% | | |
| Identified S ^**^ | | 92% ± 19% | | |  | 78% ± 9% | | |
| Identified As ^**^ | | 62% ± 19% | | |  | 86% ± 38% | | |

^a^ amount of standard added to the original sample (on an elemental basis), ^b^ percentage of added element measured after subtraction of the unspiked concentration. Standard deviations were calculated from triplicate analysis of samples. The comparison of the total elemental Se, S and As concentrations in the samples (^*^) with the elemental sum of the identified species yields the percentage of identified species (^**^).

Abbreviations: not determined (N.D.), below detection limit (<LOD), methane seleninic acid (MSeA), dimethyl selenoxide (DMSeO), methane sulfonic acid (MSA), dimethyl sulfoxide (DMSO), monomethyl arsonic acid (MMAA), dimethyl arsonic acid (DMAA), trimethyl arsine oxide (TMAO).

SUPPORTING DISCUSSION

**Formation of trapping products**

In order to explain the formation of their reaction products in the trapping experiments (Table 1), we consider average bond dissociation energies in the volatile trapped Se, S, and As molecules. It should be noted that illustrative molecular bond dissociation energies were considered, even though the exact molecular bond strengths will depend on the overall molecular structure [44]. Upon reaction with nitric acid, Se–Se and S–S bonds in DMDSe and DMDS are broken, but the C–Se and C–S bonds are maintained. This may be explained by the fact that both C–Se (234 kJ·mol^–1^) and C–S (272 kJ·mol^–1^) bonds in mono- and di-alkylated species are stronger than Se–Se (172 kJ·mol^–1^) and S–S bonds (225–251 kJ·mol^–1^) [45]. The intact methyl-groups on the Se and S atoms, both in mono- (DMDSe and DMDS) and di-methylated (DMSe and DMS) species, thus allow for deduction of the original gaseous speciation. In the investigated volatile As species, the C–As bonds (250 – 263 kJ·mol^–1^) are probably weaker than the H–As bonds in MMA and DMA (299 – 302 kJ·mol^–1^) [46]. Since non-methylated As species were found to be products of all investigated oxidation reactions, methyl groups are partially lost from MMA, DMA, and TMA in the trapping reaction, thus preventing deduction of the original gaseous speciation.

Upon cleavage of the Se–Se and S–S bond in DMDSe and DMDS, methyl-radicals may be formed analogously to observed gas phase reactions of reduced methylated S compounds with nitrate radicals [47]. Subsequently, the central Se and S atoms are oxidized and oxygen is added to the central atoms to form a thermodynamically stable compound. In the case of S, lower oxo-acids [methane sulfenic acid (S[0]) and methane sulfinic acid (S[II])] are unstable in the oxidative nitric acid medium [48,49], and MSA (S[IV]) is formed. However, MSA has also been reported as a major reaction product of the gas-phase oxidation reactions of other reduced S compounds [50]. In addition, other products have been reported from the gas-phase oxidation reaction of DMDS (e.g., sulfur dioxide, SO_2_) [51]. Although it remains unclear to what extent gas phase reactions are directly comparable with our gas trapping reactions, SO_2_ could be expected to form from complete oxidation of the central S atom. Such formation of gaseous SO_2_ could potentially explain the observed incomplete recoveries as SO_2_ would escape from the traps [47,52,53]. Finally, it should be noted that the species MSeA and MSA could be formed from the oxidation of DMSeS (a previously observed natural species) [54] [S–Se bond strength ~200 kJ·mol^–1^ (weaker than S–S bond, stronger than Se–Se bond)]. In order to guarantee the correct deduction of the original gaseous speciation from transformed oxidation products, a better understanding of the exact mechanisms of oxidation of other, naturally relevant volatile compounds in nitric acid is required.

**Chromatographic methods**

In mixed-mode chromatography, the retention of analytes on the stationary phase of the column mainly stems from ion-exchange interaction and/or reversed-phase interaction. In the ‘air method’, the mono-methylated species [MSeA (pKa unknown), MSA (pKa –1.9 [55]), and MMAA (pKa_1_ 3.6, pKa_2_ 8.7 [56])] elute before the di- or tri-methylated species [DMSeO (pKa unknown), DMSO (pKa ~35), MSM (pKa ~31), DMAA (pKa 6.2 [56]) and TMAO (pKa unknown)]. This order of elution [negatively (or more negatively) charged compounds elute before neutral (or less negatively charged) species, see Table S3 in Supporting Information File S1] may be explained by the fact that injection of the strongly acidic trapping samples generates acidic conditions on the mixed-mode column, which protonates analyte anions and increases the neutral properties of the analytes. This reduces the analyte retention based on an ion-exchange mechanism. Although the exact contributions of the retention mechanisms in the mixed-mode separation remain unknown, the dominant retention mechanism in the ‘air method’ thus most likely stems from the reversed phase exchange sites (higher logK_ow_ values increase retention). Compared to the ‘air method,’ the order of the elution of species in the ‘water method’ is almost reversed (compare Figure 3 and Figure S2 in Supporting Information File S1). Due to the prevailing slightly basic conditions of the ‘water method,’ an anion exchange separation mechanism probably dominates in this method, which is reflected by the elution of neutral species before the elution of negatively charged species (e.g., oxyanions elute last).

The slight variation in calculated LODs between individual species and between the ‘air method’ and ‘water method’ (see Table S3 in Supporting Information File S1) is likely caused by eluent-related variations in background signal, peak separation, and deviations in plasma properties at the time of elution (e.g., organic versus inorganic analytes and carbon loading of the eluent, as well as variable vapor pressure of eluting species). A minor increase in background signal (~10 min onwards, Figure S2 in Supporting Information File S1) corresponds with the increased mixing of the NH_4_NO_3_-methanol eluent and consequential nitrogen-oxygen interferences. Considering that the presented speciation methods are intended for analyzing natural samples with corresponding low environmental concentrations, the low detection limits enable reliable, simultaneous quantification of multiple species simultaneously.

SUPPORTING METHODS

**Total elemental analysis**

Analysis of the trapping liquids and aqueous samples for the total elemental concentrations of Se and As was conducted with ICP-MS, HR-ICP-MS, and ICP-OES. The total S concentrations were analyzed by ICP-OES and HR-ICP-MS. The instrumental details and detection limits for these total elemental analyses are given in Table S2 in Supporting Information File S1. For total elemental analysis, the trapping liquids were measured in a 1% HNO_3_ matrix and the aqueous samples were diluted 1:10 with ultrapure water. In both the diluted trapping liquid and the aqueous samples, 1% HPLC-grade methanol was added to enhance the signal for Se and As [57]. Inorganic Se, As, and S standards (J.T. Baker, Avantor, Griesheim, Germany) were used for calibration in all total elemental analyses. All samples and calibration standards were measured in triplicate, accompanied by in-house- (ARS-29, ARS-30, ARS-31 and ARS-32) and commercial (Merck 1631, Merck X and PRIMUS multi-anion) external standards.

**Speciation analysis**

For speciation analysis, a HPLC Dionex GP40 gradient pump (Thermo Fisher, Reinach, Switzerland) equipped with PEEK pump heads was coupled to the HR-ICP-MS. All tubing (PEEK polymer, Sigma-Aldrich, Buchs, Switzerland) was as short as reasonably possible. The injection loop volume was 20 µL. In addition to the daily instrument tuning, the HPLC-HR-ICP-MS set-up was tuned on a weekly basis with a 10 ppb Se–S solution in 2% HNO_3_ at the pump flow rate. In addition to measuring target masses of S, Se, and As, the gradient elution and inmixing of methanol was monitored on an indicator mass for carbon (^12^C^40^Ar, 51.9618 amu) in medium resolution mode at 0.8–1.25 Hz. Due to the potential interference of ^40^Ar^35^Cl on ^75^As in the medium resolution mode of HR-ICP-MS [58], care was taken that chloride did not co-elute with an As species, that the threshold concentration (>3 mg Cl·L^–1^) at which chloride yields a significant (potentially overlapping) peak was not surpassed, and that sufficient amounts of organic modifier were used to suppress the chloride interference [26].

Details of the HPLC gradients used in both the ‘air method’ and the ‘water method’ are given in Table S3 in Supporting Information File S1. Eluents were composed as follows: eluent A: 30mM NO_3_NH_4_, 1% methanol, pH 7.5, eluent B: 50mM Na_2_CO_3_-NaHCO_3_, 25% methanol, pH 8.5, and eluent C: water, pH 8.4. The eluents were prepared using ultrapure water, ultrapure HNO_3_, ultrapure ammonia, sodium bicarbonate and disodium carbonate salts, and HPLC-grade methanol. The pH was adjusted with diluted HNO_3_ or ammonia. All eluents were degassed with Ar and pre-cleaned with an Ionpac ATC 2mm ion trap column (Dionex, Thermo Fisher, Reinach, Switzerland).

Speciation analysis with the ‘air method’ was conducted on diluted (1:50 with ultrapure water) trapping liquid samples and standards in 2% HNO_3_. Eleven target analytes were investigated with the ‘air method’, including both non-volatile methylated and oxyanionic Se, S, and As species. Speciation analysis with the ‘water method’ was performed on undiluted (circumneutral) aqueous samples. Because the samples were not acidified, changes in (redox)speciation induced by acidification were prevented. In addition to the species investigated with the ‘air method’, the ‘water method’ also included Se[IV] and As[III] as target analytes. Neutral and acidified (2% HNO_3_) standards and sample dilutions were freshly prepared. Calibrations were based on a 3-point plus blank linear fit over at least a two orders of magnitude concentration range in the μg·L^–1^ range, and each of the investigated species was individually calibrated in each of the presented speciation methods. Therefore, any changes in instrumental response due to inmixing of organic eluent are accounted for by the calibration of each species at the same retention time (and thus MeOH content). An overview of the analyte retention factors, calibration ranges, correlation coefficients of the calibration curves, and limits of detection (3 × σ) is given in Table S3 in Supporting Information File S1.

**Electrospray Ionization Tandem Mass Spectrometry**

The Electrospray Ionization Tandem Mass Spectrometry (ESI-MS/MS) measurements were conducted on a Thermo LTQ Orbitrap XL ETD. The sample was introduced via a T-split with 50 µL·s^–1^ ultrapure methanol:water mixture (70:30) and 10 µL·s^–1^ sample. The trapping solutions were diluted to <0.1% HNO_3_ in order to lower the ion loading. Mass spectra were recorded in full scan mode, both in the positive and negative mode, with a mass resolution of 60,000 and a mass accuracy of <10ppm. Tandem mass spectrometry was conducted with an isolation width of 3m/z, HCD settings of 50 to 80, a mass resolution of 60,000 to 100,000 and a mass accuracy of <10ppm. In order to confirm the trapping product identities as implied from peak matching with HPLC-HR-ICP-MS, the measured spectra from experimental trapping liquids were compared with database spectra and with spectra from standard solutions of DMSO, MSA, DMSeO, and MSeA. The isotopic patterns of the measured samples and the theoretical patterns were compared in terms of accuracy as well as intensity (see Figure S1 in Supporting Information File S1). A second identification of the structure of the targeted compounds in the trapping liquids was obtained by scanning tandem mass spectrometry fragments (see Table S5 in Supporting Information File S1). However, the fragmentation of DMSO was obstructed by its low molecular mass.

SUPPORTING REFERENCES

1. Pecheyran C, Amouroux D, Donard OFX (1998) Field determination of volatile selenium species at ultra trace levels in environmental waters by on-line purging, cryofocusing and detection by atomic fluorescence spectroscopy. Journal of Analytical Atomic Spectrometry 13: 615-621.

2. Chen B, Hu B, He M, Huang Q, Zhang Y, et al. (2013) Speciation of selenium in cells byHPLC-ICP-MS after (on-chip) magnetic solid phase extraction. Journal of Analytical Atomic Spectrometry 28: 334-343.

3. De La Calle Guntinas MB, Lobinski R, Adams FC (1995) Interference-free determination of selenium(IV) by capillary gas chromatography-microwave-induced plasma atomic emission spectrometry after volatilization with sodium tetraethylborate. Journal of Analytical Atomic Spectrometry 10: 111-115.

4. Campillo N, Penalver R, Hernandez-Cordoba M, Perez-Sirvent C, Martinez-Sanchez MJ (2007) Comparison of two derivatizing agents for the simultaneous determination of selenite and organoselenium species by gas chromatography and atomic emission detection after preconcentration using solid-phase microextraction. Journal of Chromatography A 1165: 191-199.

5. Yan X-P, Sperling M, Welz B (1999) On-Line Coupling of Flow Injection Microcolumn Separation and Preconcentration to Electrothermal Atomic Absorption Spectrometry for Determination of (Ultra)trace Selenite and Selenate in Water. Analytical Chemistry 71: 4353-4360.

6. Calle Guntiñas M, Ceulemans M, Witte C, Łobiński R, Adams F (1995) Evaluation of a purge-and-trap injection system for capillary gas chromatography-microwave induced plasma-atomic emission spectrometry for the determination of volatile selenium compounds in water. Microchimica Acta 120: 73-82.

7. Duan J, Hu B (2009) Speciation of selenomethionine and selenocystine using online micro-column containing Cu(II) loaded nanometer-sized Al2O3 coupled with ICP-MS detection. Talanta 79: 734-738.

8. Vilano M, Rubio R (2000) Liquid chromatography-UV irradiation-hydride generation-atomic fluorescence spectrometry for selenium speciation. Journal of Analytical Atomic Spectrometry 15: 177-180.

9. Huang C, Hu B, He M, Duan J (2008) Organic and inorganic selenium speciation in environmental and biological samples by nanometer-sized materials packed dual-column separation/preconcentration on-line coupled with ICP-MS. Journal of Mass Spectrometry 43: 336-345.

10.Hongwei Y, Chen C, Gao Y, Li B, Chai Z (2006) Chemical Speciation Analysis of Selenium in Biological Samples by a Hyphenated Technique of High Performance Liquid Chromatography-Inductively Coupled Plasma Mass Spectrometry. Chinese Journal of Analytical Chemistry 34: 749-753.

11. Lunoe K, Skov S, Gabel-Jensen C, Sturup S, Gammelgaard B (2010) A method for analysis of dimethyl selenide and dimethyl diselenide by LC-ICP-DRC-MS. Analytical and Bioanalytical Chemistry 398: 3081-3086.

12. Zheng J, Ohata M, Furuta N (2002) Reversed-phase liquid chromatography with mixed ion-pair reagents coupled with ICP-MS for the direct speciation analysis of selenium compounds in human urine. Journal of Analytical Atomic Spectrometry 17: 730-735.

13. Muñiz-Naveiro Ó, Domínguez-González R, Bermejo-Barrera A, Bermejo-Barrera P, Cocho JA, et al. (2007) Selenium speciation in cow milk obtained after supplementation with different selenium forms to the cow feed using liquid chromatography coupled with hydride generation-atomic fluorescence spectrometry. Talanta 71: 1587-1593.

14. Gonzalez Lafuente JM, Dlaska M, Fernandez Sanchez LM, Sanz-medel A (1998) Organic and inorganic selenium speciation in urine by on-line vesicle mediated high-performance liquid chromotography-focused microwave digestion-hydride generation-inductively coupled plasma mass spectrometry. Journal of Analytical Atomic Spectrometry 13: 423-429.

15. Abbas-Ghaleb K, Gilon N, Crétier G, Mermet JM (2003) Preconcentration of selenium compounds on a porous graphitic carbon column in view of HPLC-ICP-AES speciation analysis. Analytical and Bioanalytical Chemistry 377: 1026-1031.

16. Sabe R, Rubio R, Garcia-Beltran L (2001) Selenium determination in urine with atomic fluorescence detection. Analytica Chimica Acta 436: 215-221.

17. Li KC, Shooter D (2004) Analysis of sulfur-containing compounds in ambient air using solid-phase microextraction and gas chromatography with pulsed flame photometric detection. International Journal of Environmental Analytical Chemistry 84: 749-760.

18. Leck C, Baagander LE (1988) Determination of reduced sulfur compounds in aqueous solutions using gas chromatography-flame photometric detection. Analytical Chemistry 60: 1680-1683.

19. Gerbersmann C, Lobinski R, Adams FC (1995) Determination of volatile sulfur compounds in water samples, beer and coffee with purge-and-trap gas-chromatography microwave-induced plasma atomic emission spectrometry. Analytica Chimica Acta 316: 93-104.

20. Kaasalainen H, Stefánsson A (2011) Chemical analysis of sulfur species in geothermal waters. Talanta 85: 1897-1903.

21. Luther III GW, Church TM, Powell D (1991) Sulfur speciation and sulfide oxidation in the water column of the Black Sea. Deep Sea Research Part A Oceanographic Research Papers 38, Supplement 2: S1121-S1137.

22. Ojala M, Ketola R, Mansikka T, Kotiaho T, Kostiainen R (1997) Detection of volatile organic sulfur compounds in water by headspace gas chromatography and membrane inlet mass spectrometry. HRC-Journal of High Resolution Chromatography 20: 165-169.

23. Lin L-Y, Jiang S-J (2009) Determination of Sulfur Compounds in Water Samples by Ion Chromatography Dynamic Reaction Cell Inductively Coupled Plasma Mass Spectrometry. Journal of the Chinese Chemical Society 56: 967-973.

24. Divjak B, Goessler W (1999) Ion chromatographic separation of sulfur-containing inorganic anions with an ICP-MS as element-specific detector. Journal of Chromatography A 844: 161-169.

25. Lestremau F, Desauziers V, Roux JC, Fanlo JL (2003) Development of a quantification method for the analysis of malodorous sulphur compounds in gaseous industrial effluents by solid-phase microextraction and gas chromatography-pulsed flame photometric detection. Journal of Chromatography A 999: 71-80.

26. Ammann AA (2010) Arsenic speciation by gradient anion exchange narrow bore ion chromatography and high resolution inductively coupled plasma mass spectrometry detection. Journal of Chromatography A 1217: 2111-2116.

27. Bednar AJ, Garbarino JR, Burkhardt MR, Ranville JF, Wildeman TR (2004) Field and laboratory arsenic speciation methods and their application to natural-water analysis. Water Research 38: 355-364.

28. Ali I, Aboul-Enein HY (2002) Speciation of arsenic and chromium metal ions by reversed phase high performance liquid chromatography. Chemosphere 48: 275-278.

29. Chu Y-L, Jiang S-J (2011) Speciation analysis of arsenic compounds in edible oil by ion chromatography–inductively coupled plasma mass spectrometry. Journal of Chromatography A 1218: 5175-5179.

30. Xie R, Johnson W, Spayd S, Hall GS, Buckley B (2006) Arsenic speciation analysis of human urine using ion exchange chromatography coupled to inductively coupled plasma mass spectrometry. Analytica Chimica Acta 578: 186-194.

31. Chen Z, Akter KF, Rahman MM, Naidu R (2008) The separation of arsenic species in soils and plant tissues by anion-exchange chromatography with inductively coupled mass spectrometry using various mobile phases. Microchemical Journal 89: 20-28.

32. Wrobel K, Wrobel K, Parker B, Kannamkumarath SS, Caruso JA (2002) Determination of As(III), As(V), monomethylarsonic acid, dimethylarsinic acid and arsenobetaine by HPLC–ICP–MS: analysis of reference materials, fish tissues and urine. Talanta 58: 899-907.

33. Yan X-P, Kerrich R, Hendry MJ (1998) Determination of (Ultra)trace Amounts of Arsenic(III) and Arsenic(V) in Water by Inductively Coupled Plasma Mass Spectrometry Coupled with Flow Injection On-Line Sorption Preconcentration and Separation in a Knotted Reactor. Analytical Chemistry 70: 4736-4742.

34. Planer-Friedrich B, Lehr C, Matschullat J, Merkel BJ, Nordstrom DK, Sandstrom MW (2006) Speciation of volatile arsenic at geothermal features in Yellowstone National Park. Geochimica et Cosmochimica Acta 70: 2480-2491.

35. Wickenheiser EB, Michalke K, Drescher C, Hirner AV, Hensel R (1998) Development and application of liquid and gas-chromatographic speciation techniques with element specific (ICP-MS) detection to the study of anearobic arsenic metabolism. Fresenius Journal of Analytical Chemistry 362: 498-501.

36. Martı́nez-Bravo Y, Roig-Navarro AF, López FJ, Hernández F (2001) Multielemental determination of arsenic, selenium and chromium(VI) species in water by high-performance liquid chromatography–inductively coupled plasma mass spectrometry. Journal of Chromatography A 926: 265-274.

37. Niedzielski P (2005) The new concept of hyphenated analytical system: Simultaneous determination of inorganic arsenic(III), arsenic(V), selenium(IV) and selenium(VI) by high performance liquid chromatography–hydride generation–(fast sequential) atomic absorption spectrometry during single analysis. Analytica Chimica Acta 551: 199-206.

38. Peachey E, Cook K, Castles A, Hopley C, Goenaga-Infante H (2009) Capabilities of mixed-mode liquid chromatography coupled to inductively coupled plasma mass spectrometry for the simultaneous speciation analysis of inorganic and organically-bound selenium. Journal of Chromatography A 1216: 7001-7006.

39. Afton S, Kubachka K, Catron B, Caruso JA (2008) Simultaneous characterization of selenium and arsenic analytes via ion-pairing reversed phase chromatography with inductively coupled plasma and electrospray ionization ion trap mass spectrometry for detection: Applications to river water, plant extract and urine matrices. Journal of Chromatography A 1208: 156-163.

40. Feldmann J (1997) Summary of a calibration method for the determination of volatile metal(loid) compounds in environmental gas samples by using gas chromatography-inductively coupled plasma mass spectrometry. Journal of Analytical Atomic Spectrometry 12: 1069-1076.

41. Diaz-Bone RA, Hollmann M, Wuerfel O, Pieper D (2009) Analysis of volatile arsenic compounds formed by intestinal microorganisms: rapid identification of new metabolic products by use of simultaneous EI-MS and ICP-MS detection after gas chromatographic separation. Journal of Analytical Atomic Spectrometry 24: 808-814.

42. Campillo N, Peñalver R, López-García I, Hernández-Córdoba M (2009) Headspace solid-phase microextraction for the determination of volatile organic sulphur and selenium compounds in beers, wines and spirits using gas chromatography and atomic emission detection. Journal of Chromatography A 1216: 6735-6740.

43. Meija J, Montes-Bayón M, Le Duc DL, Terry N, Caruso JA (2002) Simultaneous monitoring of volatile selenium and sulfur species from se accumulating plants (wild type and genetically modified) by GC/MS and GC/ICPMS using solid-phase microextraction for sample introduction. Analytical Chemistry 74: 5837-5844.

44. Solomons GTW, Fryhle CB (2007) Organic Chemistry: Wiley.

45. Cottrell TL (1958) The Strengths of Chemical Bonds. London: Butterworths.

46. Mestrot A, Merle JK, Broglia A, Feldmann J, Krupp EM (2011) Atmospheric Stability of Arsine and Methylarsines. Environmental Science & Technology 45: 4010-4015.

47. Jensen NR, Hjorth J, Lohse C, Skov H, Restelll G (1992) Products and mechanisms of the gas phase reactions of NO3 with CH3SCH3, CD3SCD3, CH3SH and CH3SSCH3. Journal of Atmospheric Chemistry 14: 95-108.

48. Carvalho NF, Silva SP, Resende SM (2011) Theoretical determination of the properties of Methanesulfinic and Methanesulfonic acids. Journal of the Brazilian Chemical Society 22: 950-954.

49. Paula Silva S, Maris Resende S (2011) Thermochemical Study of the Reactions of Methanesulfinic acid with NO, NO3 and O2. Simposio Brasileiro de Quimica Teorica. Ouro Preto, MG, Brasil.

50. Yin FD, Grosjean D, Seinfeld JH (1986) Analysis of atmospheric photooxidation mechanisms for organosulfur compounds. Journal of Geophysical Research-Atmospheres 91: 14417-14438.

51. Yin FD, Grosjean D, Flagan RC, Seinfeld JH (1990) Photooxidation of dimethylsulfide and dimethyldisulfide 2. Mechanism evaluation. Journal of Atmospheric Chemistry 11: 365-399.

52. Atkinson R, Aschmann SM, Hasegawa D, Thompsoneagle ET, Frankenberger WT (1990) Kinetics of the atmospherically important reactions of dimethyl selenide. Environmental Science & Technology 24: 1326-1332.

53. Atkinson R, Pitts JN, Aschmann SM (1984) Tropospheric reactions of dimethyl sulfide with nitrogen oxide (NO3) and hydroxyl radicals. The Journal of Physical Chemistry 88: 1584-1587.

54. Amouroux D, Pecheyran C, Donard OFX (2000) Formation of volatile selenium species in synthetic seawater under light and dark experimental conditions. Applied Organometallic Chemistry 14: 236-244.

55. Guthrie JP (1978) Hydrolysis of esters of oxyacids: pKa values for strong acids. Canadian Journal of Chemistry 56: 2342-2354.

56. Francesconi KA, Kuehnelt, D. (2002) Arsenic compounds in the environment. In: Frankenberger Jr. WT, editor. Environmental Chemistry of Arsenic. New York: Marcel Dekker Inc. pp. 51-94.

57. Larsen EH, Sturup S (1994) Carbon-enhanced inductively-coupled plasma-mass spectrometric detection of arsenic and selenium and its application to arsenic speciation. Journal of Analytical Atomic Spectrometry 9: 1099-1105.

58. Ammann A (2011) Arsenic Speciation Analysis by Ion Chromatography - A Critical Review of Principles and Applications. American Journal of Analytical Chemistry 2: 27-45.
